# Editorial: Methods in motor neuroscience

**DOI:** 10.3389/fnhum.2026.1788976

**Published:** 2026-02-03

**Authors:** Ricardo Antonio Salido-Ruiz

**Affiliations:** Centro Universitario de Ciencias Exactas e Ingenierías (CUCEI), Universidad de Guadalajara (UDG), Guadalajara, Mexico

**Keywords:** brain plasticity, motor planning and execution, Parkinson's disease, sliding techniques, tDCS

Motor neuroscience has experienced rapid methodological growth in recent years. This is because the need to better characterize the neural, biomechanical, and behavioral mechanisms underlying motor control in both: healthy and clinical populations. The Research Topic “*Methods in Motor Neuroscience*” was precisely conceived to bring together innovative methodological approaches that expand our ability to measure, analyze, and modulate motor behavior across multiple levels of observation.

These six articles that were included in this Research Topic collectively highlight the diversity of contemporary methodological strategies in motor neuroscience, spanning biomechanical analysis, motion tracking technologies, neuromodulation techniques, advanced electroencephalographic (EEG) modeling, and multimodal neuroimaging. All these contributions underscore the importance of methodological rigor and innovation as foundational elements for advancing both: basic and translational motor neuroscience research. Next we describe some of the investigations of these contributions.

Hua et al. investigated the effects of different sliding techniques on lower limb biomechanics and muscle synergy during curling delivery, with a particular focus on joint kinetics and muscular coordination analyzed from kinematic and electromyographic (EMG) data. By combining biomechanical modeling with muscle synergy analysis, this work provides valuable insight into how variations in motor technique shape neuromuscular control strategies.

Vyazmin et al. introduced a comprehensive experimental framework to study motor planning and execution using 3D-printed objects combined with motion tracking technology more precisely High-resolution kinematic data were captured using an infrared motion tracking system. Their paradigm enables precise temporal and spatial separation of planning and execution phases, addressing a long-standing methodological challenge in motor neuroscience.

Wójcik et al. explored the effects of a single session of transcranial direct current stimulation (tDCS) of the motor cortex on the left side combined with mirror therapy on hand function in healthy individuals. By integrating neuromodulation with sensorimotor feedback paradigms, this work contributes to the growing methodological literature examining how non-invasive brain stimulation can modulate motor performance.

Two contributions focused on EEG-based methodologies applied to Parkinson's disease. Kia et al. employed Koopman-based linearization to model preparatory EEG dynamics during galvanic vestibular stimulation, providing a mathematically grounded approach for capturing non-linear neural dynamics. Complementarily, Alizadeh et al. examined EEG dynamical features during variable-intensity cycling exercise, highlighting how exercise intensity modulates neural activity patterns.

Finally, Zhang et al. investigated brain plasticity associated with prolonged shooting training using a multimodal neuroimaging approach. This study provides a comprehensive view of training-induced neural adaptations and underscores the value of multimodal methodologies for understanding motor learning and plasticity.

Collectively, the contributions of this Research Topic emphasize the importance of integrative, methodologically robust approaches in motor neuroscience. Future research will benefit from continued multimodal integration, advanced analytical techniques, and expanded application to diverse clinical populations.

